# Interocular yoking in human saccades examined by mutual information analysis

**DOI:** 10.1186/1753-4631-4-S1-S10

**Published:** 2010-06-03

**Authors:** Masaki Maruyama, Peter BC Fenwick, Andreas A Ioannides

**Affiliations:** 1 Laboratory for Human Brain Dynamics, RIKEN Brain Science Institute, 2-1 Hirosawa, Wakoshi, Saitama 351-0198, Japan; 2 Present address: CEA/DSV/I2BM / NeuroSpin, INSERM U992 - Cognitive Neuroimaging Unit, Bât 145 - Point Courrier 156, Gif sur Yvette F-91191, France; 3 Laboratory for Human Brain Dynamics. AAI Scientific Cultural Services Ltd., 33, Arch. Makarios III Avenue, Nicosia, 1065, Cyprus

## Abstract

**Background:**

Saccadic eye movements align the two eyes precisely to foveate a target. Trial-by-trial variance of eye movement is always observed within an identical experimental condition. This has often been treated as experimental error without addressing its significance. The present study examined statistical linkages between the two eyes’ movements, namely interocular yoking, for the variance of eye position and velocity.

**Methods:**

Horizontal saccadic movements were recorded from twelve right-eye-dominant subjects while they decided on saccade direction in Go-Only sessions and on both saccade execution and direction in Go/NoGo sessions. We used infrared corneal reflection to record simultaneously and independently the movement of each eye. Quantitative measures of yoking were provided by mutual information analysis of eye position or velocity, which is sensitive to both linear and non-linear relationships between the eyes’ movements. Our mutual information analysis relied on the variance of the eyes movements in each experimental condition. The range of movements for each eye varies for different conditions so yoking was further studied by comparing GO-Only vs. Go/NoGo sessions, leftward vs. rightward saccades.

**Results:**

Mutual information analysis showed that velocity yoking preceded positional yoking. Cognitive load increased trial variances of velocity with no increase in velocity yoking, suggesting that cognitive load may alter neural processes in areas to which oculomotor control is not tightly linked. The comparison between experimental conditions showed that interocular linkage in velocity variance of the right eye lagged that of the left eye during saccades.

**Conclusions:**

We conclude quantitative measure of interocular yoking based on trial-to-trial variance within a condition, as well as variance between conditions, provides a powerful tool for studying the binocular movement mechanism.

## Background

Saccadic eye movements are rapid shifts of gaze onto a new point in the visual field so that the image of the object at that point falls on the fovea. When the two eyes foveate the same object, visual performance is improved in dim light [1], and the two eyes allow stereopsis through detailed comparison of the retinal images from the two eyes. The linking together of the movement of the two eyes, usually by opposite muscle pairs is ‘yoking’, one of the simplest statements of *Herings Law* or the law of equal innervation. With horizontal saccades to the left the yoked pair are predominantly the lateral rectus of the left eye and medial rectus of the right. Yoking during saccades has to be maintained in the presence of an asymmetry of muscle strength and media stiffness. Collins et al [2], have shown that for maintaining extreme horizontal gaze the medial rectus developed a force 26% greater than the lateral rectus. There is also an asymmetry of the media, as the stiffness of tissues restraining globe motion for the adducting eye was 11% greater than for the eye in the abducting movement.

When a subject makes saccades from an identical fixation point to an identical target, we always observe variation of eye movements among the saccades. This trial-to-trial variation has often been treated as just experimental noise and cancelled out by averaging over trials. However, Erkelens and Sloot [3] indicated by the often-used correlation coefficient analysis that statistical relatedness in trial-to-trial variance is strong between the eyes’ saccade directions. A further paper looked at trial to trial variation in peak velocity, saccade duration, and curvature (angular velocity over time) and noted that high variances in these measures were compatible with saccade accuracy. They found the interocular correlation of trial-to-trial variance was also high for peak velocity, saccade duration and saccade curvature [4]. Their findings suggested the presence of a common saccade generator for the eyes, which fluctuates among trials. They also commented that assuming a local feedback loop was guiding saccades then the high correlation between duration and curvature raised the possibility of common feed back from the two eyes. Their finding implies that intensive analysis of variance will yield new insight into interocular yoking. In addition extracting maximum information from limited sampled data could be valuable in clinical environments because a short time for eye movement recording reduces the stress for patients.

In this study we set out to examine interocular yoking in horizontal saccades by looking at the variance of movement for each eye across trials. The degree of yoking was quantified by the technique of Mutual Information (MI) analysis [5] which unlike correlation coefficient analysis, is sensitive to both linear and non-linear relationships between the eyes movements. We wanted to know if MI analysis might provide new evidence for the functioning of the oculomotor system beyond that given by classical correlation coefficient analysis. We also used experimental differences in the averages and standard deviations (SD) amongst trials for each eye. The averages and SDs modulated by a common motor driver for both eyes could also indicate the course of interocular yoking.

Although eye position in horizontal saccades is a simple integration of velocity in each trial [6], non-linear calculation of MI analysis over trials could in principle provide different aspects of how position and velocity are controlled. Therefore we compared the yoking in the eyes’ positions with respect to velocity.

Finally we wanted to know if the degree of yoking can assist studies on the effects of cognitive load. Our previous magnetoencephalographic (MEG) study in humans observed an increase in interactive activity between the brainstem and cerebellum during saccades with a high cognitive-load task [7]. In the current study, we used a subset of the identical saccade tasks used in the MEG study and examined whether altering the cognitive load influences the degree of yoking.

## Methods

### Subjects

Twelve healthy right-handed men participated in the experiment. Their ages ranged from 19 to 39 years, with a mean age of 27 years. All subjects had right eye dominance, normal visual acuity and had neither ophthalmological nor neurological abnormalities. Right-handedness was determined by the 10-item Edinburgh handedness inventory [8] and eye dominance with the near-far alignment eye test. Six subjects were Japanese fluent in up/down reading and the others were Caucasian with little or no Japanese reading skills. Before starting the experiment, the protocol and the recording method of eye movements were explained, and participants gave informed consent. All procedures were approved by the RIKEN Ethical Committee.

### Oculomotor task

**Figure 1:**
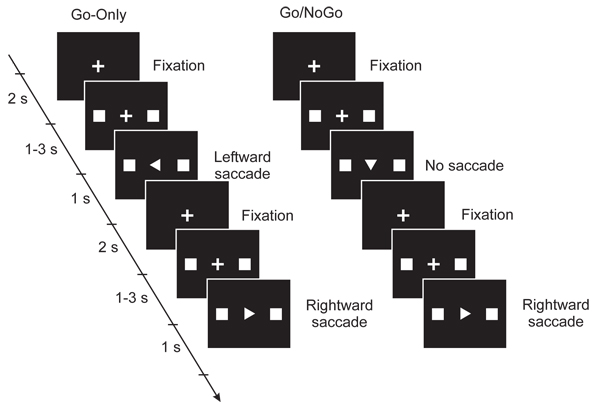
**Schematic representations of the stimulus display sequence and the oculomotor task in the Go-Only sessions and in the Go/NoGo sessions.** Every trial started with the fixation of a small central cross. Two squares, targets for the saccade, were then presented simultaneously on both left and right sides of the fixation cross. The direction of the saccade was shown by the pointing of an arrowhead which then appeared in place of the fixation cross. In the Go-Only sessions, the subjects made a saccade to one target in each trial. The Go/NoGo sessions contained trials in which the arrowhead pointed downward instructing the subjects to hold the fixation.

Different tasks had different cognitive load, depending on how the information about the movements and directions were given to the subject. Every trial started with the illumination of a fixation point, a small cross, at the centre of the screen (Fig. 1). While the subjects maintained fixation, two squares were simultaneously illuminated on the horizontal axis to the left and right of the fixation point at an angular distance of 12 deg. After an interval of 1 – 3 s, an arrowhead was displayed at the fixation point. The arrowhead’s direction indicated which square was to be a target for the saccade. For the Go trial the subjects made a saccade towards the target as soon as they could after the appearance of the arrowhead and then fixated the target until the squares disappeared and the fixation cross reappeared in the centre of the screen. In the NoGo trial, if the direction of the arrowhead was downward, they were instructed to keep their fixation at the centre and not to make a saccade. The trial ended and the next trial began when the central fixation point reappeared. The arrowhead always contained information about the direction to move which was not available before the trial. The degree of cognitive load was increased in a Go/NoGo trial when the subject had to process the Go/NoGo information.

Each subject had 12 sessions on two consecutive days lasting about one hour for each day. Half of the sessions contained only Go trials (Go-Only session) and the other half contained Go and NoGo trials (Go/NoGo session). Each session contained five leftward and five rightward saccade trials in the Go-Only session and five extra NoGo trials in the Go/NoGo session. Thus Go-Only and Go/NoGo sessions consisted of 10 and 15 trials, respectively. These two types of sessions were alternated and in each session the left and right directions were randomized. Before starting each session, the subjects were informed which session was to follow. The total number of trials was 30 for each saccade task, leftward, rightward in Go-Only (total 60) and an additional 30 NoGo trials in Go/NoGo (total 90). The session types were Go-Only, Go/NoGo and experiment days were 1^st^ and 2^nd^.

### Visual stimuli

The visual stimuli were presented on a 21-in. flat CRT display placed 58 cm distant from the subjects. The squares, arrowheads and the fixation cross subtended 0.85 deg. The display had a frame refresh rate of 85 Hz. The stimuli were generated by Presentation software (Neurobehavioral systems, Inc., version 0.80, Albany, CA, USA).

### Recording and preprocessing

Eye movements were recorded with an infrared reflection system (Takei; TKK2901). Before starting each session, the system was calibrated by asking the subjects to fixate several times at 12 positions around the edge of a square (24 × 24 deg) and at the center. This procedure was repeated until the calibration was adequately performed. The subject’s head was stabilized by supports for the chin and forehead. Eye position was recorded from both eyes independently and simultaneously at a frequency of 1000 Hz. The system had a noise level of 0.13 deg. The horizontal eye position and the image signals were stored on a computer for off-line analyses. Eye velocity was calculated by off-line differentiation of the position records between neighboring time slices.

Our approach was to characterize each saccade by three latency features. The first feature defined the start of the saccade and it is called the saccade initiation time. The second feature corresponded to the latency with maximum eye velocity and it is called the peak-velocity time. The third feature defined the end of the saccade and it is called the saccade end time. These parameters were defined automatically based on eye velocity criteria. For the details of the automatic procedures, please see the Supplementary Materials in Additional file 1. The initiation time was used to align the saccades’ data over the trials for the analyses of average and standard deviation (SD), and also for MI and correlation coefficient analyses.

Eye positions and velocities for each trial were segmented for 50 ms before and 100 ms after the detected saccadic initiation. Trials were excluded that contained too early onset, <150 ms after the start of the cue image, or too late >800 ms, or a saccade toward the opposite direction, or were contaminated by blinks. Between 9 and 30 trials were obtained for each saccade direction, session type, experiment day and subject (25 trials on average).

### Statistical analysis of average eye movement

Each eye can show different movements by varying the experimental conditions (leftward vs. rightward, Go-Only vs. Go/NoGo, 1^st^ day vs. 2^nd^ day). The differential effect of the condition on the eyes indicates the yoking. We first tested each condition’s effect on each eye’s velocity by the examination of the velocity from each time slice using an analysis of variance (ANOVA) SPSS 15.0 (SPSS Inc., Chicago, Illinois, USA). We identified significant effects in the velocity that were common between the eyes. The ANOVA was set up to examine differences in *average* velocity, thus this approach showed yoking based on the average velocity changes following the different experimental conditions.

Because of the number of slices analysed many Type 1 errors were expected to occur by chance, so the effects were defined only as significant when they continuously satisfied the criterion for a Type 1 error of p<0.05 for 10 ms or more. When interactions were significant, post-hoc multiple comparisons were performed using Scheffe’s procedure. An identical method was also used for the analysis of eye positions.

### Mutual information analysis

In MI analysis, the key measurement for quantification of relationships among variables is entropy [5]. For example, the entropy of the left eye can be partitioned into *N_LE_* possible states defined as

,	(1)

where *p_LE_(i)* is the probability that the left eye will be in state *i* and * p_LE_(i)* = 1. The entropy for events of the right eye can be correspondingly defined as

.	(2)

Simultaneous recordings of the two eyes’ movements, as in this study, allowed the measurement of the joint probability, *p(i,j)*, that the left and right eyes will be in state *i* and *j*. The entropy of joint events is

,	(3)

where *ΣΣ p(i,j)* = 1. If statistical interactions or common influences exist between the two eye’s movements, i.e., *p(i,j) ≠ p_LE_(i)p_RE_(j)*, information on the right (left) eye’s state can reduce the uncertainty on the left (right) eye’s state, which can be represented by a reduction of the joint entropy. The MI quantifies this reduction as

.	(4)

The MI vanishes only if the two eye’s states are completely independent. In this study, eye velocity and position served as measures of ocular state for the purpose of MI analysis. We analyzed two relationships: between left eye velocity and right eye velocity (velocity MI) and between left eye position and right eye position (positional MI).

The joint probability was estimated from the paired samples by the following procedure. First, the position and velocity of each trial were aligned to the timing of saccade initiation. Next, a short analysis window of 11 ms was set for the left and right eye between –50 and 100 ms from the initiation of the saccade. The samples within the windows were paired between the eyes. Then, the joint probability distribution was estimated by applying a Gaussian kernel to the paired samples. The smoothing length of the kernel was optimized, based on the samples, using a likelihood cross validation method [9]. This is a completely automatic method for choosing the smoothing length. It minimizes the difference between the estimated probability distribution and the true one in terms of information distance that has been constructed from the samples. For details of the optimized Gaussian kernel method, please see the Supplementary Materials in Additional file 1. Finally, MI was calculated by using Eq. (1) ~ (4). The analysis window was shifted in steps of 5 ms, which gave enough time resolution to find out fluctuation in yoking during the saccade.

Measurements of MI can overestimate weak relationship because of limited sample sizes. We therefore estimated the overestimation by a randomization procedure. The pairs of the two eyes’ data were randomized across trials and across time slices within the analysis window, MI was then calculated in the same way described above for 100 times and averaged, producing the mean MI of the randomized data (MI_Ran_). In all MI results, we used the corrected MI value, MI_Cor_ defined as the difference between the MI value obtained from the original data, MI_Ori_ and the mean across the 100 randomizations: MI_Cor_ = MI_Ori_ – MI_Ran_. The preliminary simulation with computer generated samples confirmed that this removed the overestimation and also showed a reduction in the standard deviation when there was no statistical relationship (Supplementary Fig. 1A and B in Additional file 2).

The association of velocity MI with positional MI was studied using the differences between the experimental conditions. This was tested by an ANOVA (direction × session type × day × subject). The effects were defined as significant only when they continuously satisfied a criterion for Type 1 error of p<0.05 for 10 ms or more. The latencies for significant differences in velocity MI with respect to those in positional MI were compared.

The MI estimation was influenced by sample size. Smaller sample sizes produced lower MI estimations (Supplementary Fig. 1C in Additional file 2). While there was the possibility of significant MI differences derived only from the sample size difference, the ANOVA set up to show such changes did not find any significant difference in sample size between the conditions (p > 0.05). Although the different sample sizes increased the standard deviation of MI among the subjects and thus decreases the sensitivity of the ANOVA, there were sufficient significant differences for the association between velocity and positional MI to support the validity of this analysis method.

Spearman’s rank correlation coefficient (CR) analysis was also included in order to be compared with MI analysis and highlight any of MI’s advantages or disadvantages. The preparation of the paired samples and the statistical test for CR analysis were identical to those for the MI analysis.

### Eye movement variances

MI analysis relied on the variance of the eyes movements to show the interocular statistical relationships. A decrease of MI could reflect both a variance decrease and/or a weak relationship in yoking between the eyes. To distinguish the two cases, we examined the SD of samples from each eye among the trials and within the analysis window, set in steps of 5 ms. The differences in SD between the experimental conditions were tested for each eye by ANOVA. The effects were significant when they satisfied the criterion of Type 1 error of p<0.05 for 10 ms or more.

Any common significant differences in SD between the eyes were also used to show the interocular relationships, as was done for the differences in the averages. The ANOVA was set up to examine differences in the *variance* of the eyes’ movements, thus this approach showed yoking based on variance changes following the different experimental conditions.

## Results

### Latencies of saccades

**Figure 2:**
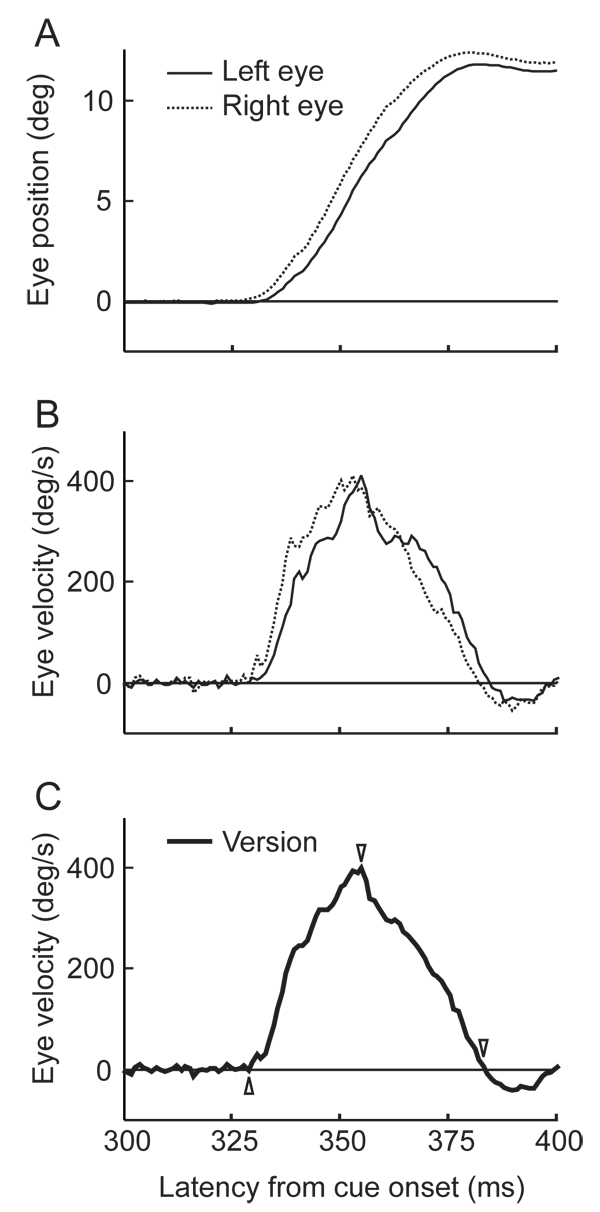
**A, B. Example of different time courses in position and velocity between the left eye (solid lines) and the right eye (dotted lines)**, obtained from a single trial in which the saccadic target was on the right side. Positive deflections shown on the vertical axis of A, B and C denote rightward movements, and thus indicate the eye movement toward the target. **C.** Version velocity, i.e., the averaged velocity between the eyes. The saccade initiation, peak-velocity and end of the trial are indicated by arrowheads, based on the version velocity. Horizontal axis is taken from the onset of the arrowhead cue image (see Fig. 1).

Figure 2 shows representative eye position and velocity obtained from one single trial of rightward saccade (1^st^ day, GoOnly session). During the saccade from the center (0 deg) to the target position (12 deg), the right eye (dotted line in Fig. 2A) led the left eye (solid line). The eye velocities in Fig. 2B also show that the right eye’s saccade was slightly earlier with respect to the left eye. This result is consistent with previous studies in which the abducting saccades led the adducting ones [10-12]. In figure 2C, the arrow heads indicate the time at initiation, peak-velocity and the end in this trial, given by the average velocity between the eyes.

**Figure 3:**
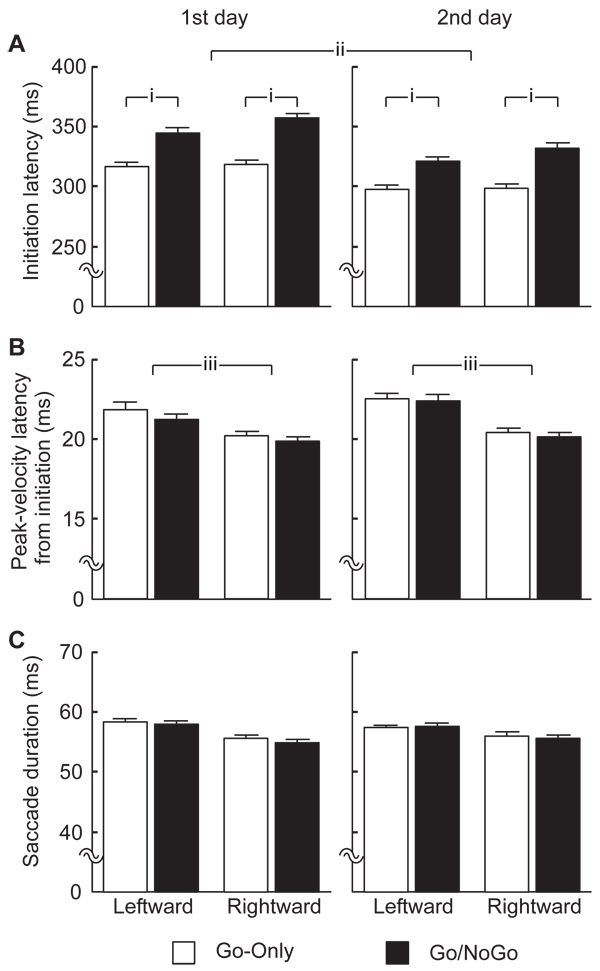
**Averaged times** for: **A.** Saccade initiation from the onset of cue image; **B.** Peak-velocity from the saccade initiation; **C.** Saccade duration. The average was performed separately for the 1^st^ day (left panels) and the 2^nd^ day (right panels), for the Go-Only sessions (open bars) and the Go/NoGo sessions (filled bars), for the leftward saccades (left paired-bars in each panel) and the rightward saccades (right paired-bars). Error bars represent ± 1 SE. The saccades initiated significantly earlier: *i.* in the Go-Only than in the Go/NoGo sessions (p<0.001); *ii.* on the 2^nd^ day than on the 1^st^ day (p<0.001); *iii.* The peak-velocity was significantly earlier for the rightward than the leftward saccades (p<0.05).

	Initiation times from the onset of the cue image were compared between saccade directions, session types and days in Fig. 3A. A four-way ANOVA (direction × type × day × subject) shows that the initiation times in the Go-Only sessions were significantly shorter than in the Go/NoGo sessions (*i* in Fig. 3A; F_1,12_=249, p<0.001). The initiation times on the 2^nd^ day were significantly shorter than on the 1^st^ day (*ii*; F_1,11_=20.4, p<0.001), representing a practice effect. A significant interaction between the direction effect and the type effect was found (F_1,12_=6.14, p<0.05). Next, peak-velocity latency from the time of initiation were examined. The ANOVA showed that latencies of the peak velocity of the rightward saccades, mean 20 ms, were significantly shorter than for leftward ones (22 ms) (*iv* in Fig. 3B; F_1,11_=4.92, p<0.05). The length of saccade duration obtained by subtraction of the initiation time from the end time was studied (Fig. 3C). The ANOVA did not show any significant differences (p>0.05) except for individual variances. The grand average of the duration was 57 ms. Hereafter, we use the average latencies of the peak velocity (21 ms) and the end (57 ms) for the reference of saccadic key events.

### Average eye velocities

**Figure 4:**
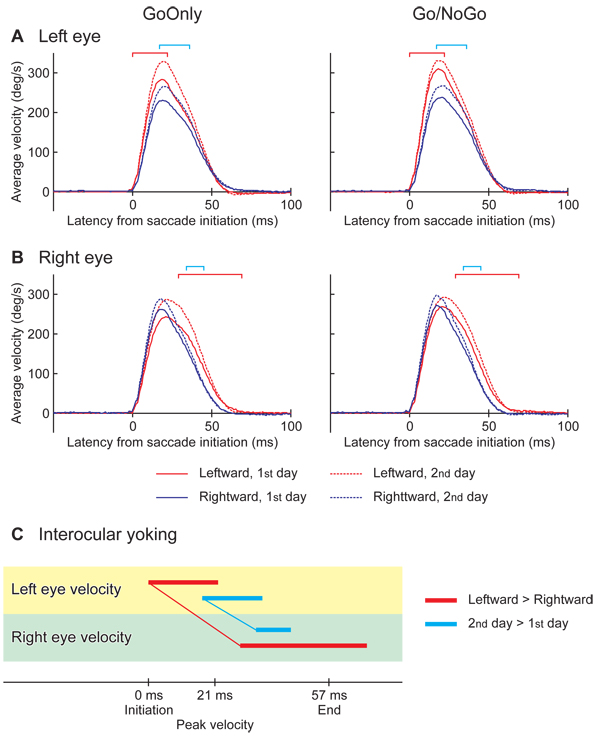
**Dependence of averaged eye velocities on experimental conditions:A.** Left eye; **B.** Right eye. Averages among all subjects are shown for the saccade toward the left (red lines) and right (blue lines) in the sessions of Go-Only (left panels) and Go/NoGo (right panels), on the 1^st^ day (solid lines) and 2^nd^ day (dashed lines). Positive deflections of velocity of eye movements toward saccadic target. Horizontal axes are the latency from the saccade initiation. The latencies of significantly faster movements are denoted by red square brackets for the leftward saccade relative to the rightward, and by cyan square brackets for the saccades on the 2^nd^ day than 1^st^ day. The significant differences were revealed by ANOVA factoring the saccade direction, the session type, the experiment day and the subject (p < 0.05 for 10 ms or more). **C.** Time courses comparisons between the eyes based on the significant differences. The latencies of significant difference with respect to the saccade directions are indicated by red horizontal bars. The onsets of the significant difference are linked between the eyes by a thin red line, showing eye yoking. Cyan horizontal bars linked by a thin line indicated the yoking found based on the significant day effect.

	The averages of eye velocity over all subjects are shown for the left eye in Fig. 4A. Positive deviations denote eye movements toward the saccadic target for either leftward or rightward movements. The left eye velocity of leftward saccades (red lines) was significantly higher relative to the rightward ones (blue lines) in a period from the initiation to the peak velocity (between 0 and 22 ms from the saccade initiation; red square brackets), as shown by an ANOVA (direction × type × day × subject) for each time slice. Likewise an ANOVA for the right eye found a significant difference in velocity between the directions, but this was after the peak velocity (between 29 and 69 ms; red square brackets in Fig. 4B). The ANOVAs also identified latencies of significantly higher velocities on the 2^nd^ day (dashed lines) relative to the 1^st^ day (solid lines) in both eyes (left eye: between 17 and 36 ms, right eye: between 34 and 45 ms; cyan square brackets), representing a practice effect. The onsets of significant differences are linked between the eyes in Fig. 4C. Both effects of direction (red line) and day (cyan line) were earlier in the left eye.

	ANOVAs for position found significant differences between the abducting and adducting saccades, between the Go-Only and Go/NoGo sessions in both eyes and between the days for the left eye (Supplementary Fig. 2 in Additional file 3). The leading eye based on the direction effect (right eye leading) was not consistent with that based on the session type effect (left eye leading).

### Variances of eye velocities

**Figure 5:**
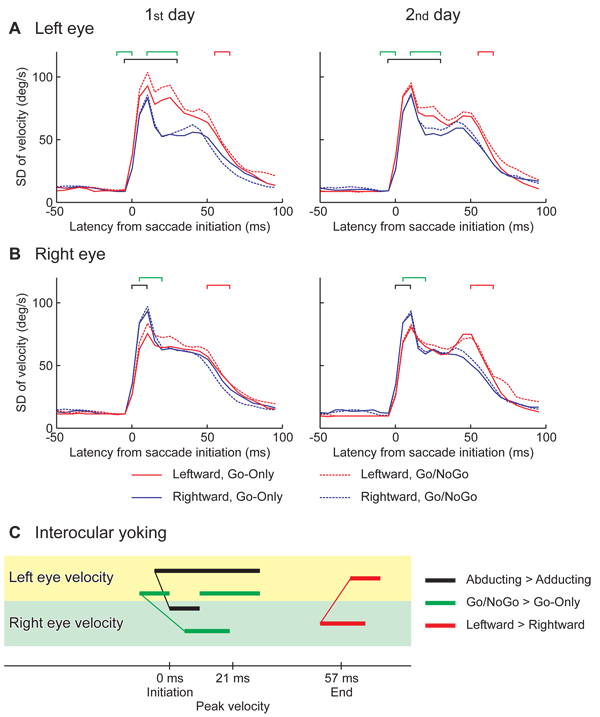
**Trial-to-trial variances related to eye velocities and experimental conditions:A.** Left eye; **B.** Right eye. Standard deviation (SD) within each subject is averaged among all subjects for the saccade toward the left (red lines) and right (blue lines) for the sessions of Go-Only (solid lines) and Go/NoGo (dashed lines), on the 1^st^ day (left panels) and 2^nd^ day (right panels). Horizontal axes are the latency from saccade initiation. The latencies of significantly larger variances are denoted by black square brackets for the abducting saccade relative to the adducting, by red square brackets for the leftward saccade relative to the rightward, and by green square brackets for the saccades in the Go/NoGo sessions than the Go-Only. The significant differences were calculated by ANOVA factoring the saccade direction, the session type, the experiment day and the subject (p < 0.05 for 10 ms or more). To see the difference between the Go/NoGo and Go-Only sessions, see Supplementary figure 3A and B in Additional file 3. **C.** Time course comparisons between the eyes were based on the significant differences. The latency of significant differences between the abducting and adducting saccades are shown by black horizontal bars. The onsets of the significant dependencies are linked between the eyes by a thin black line, showing eye yoking. Linked horizontal bars in red indicate the yoking found based on the significant difference between the leftward and rightward saccades, and that in green showed the significant effect of cognitive load.

	The SD of eye velocity is shown for the left eye in Fig. 5A and for the right eye in 5B. Averages of SDs over all subjects are shown for each experimental condition. The SD increased steeply at the initiation of the saccade and decreased after 15 ms, and then increased again in some conditions before the end of the saccade.

An ANOVA for the left eye’s velocity found significantly larger variance in the leftward saccades (red lines) than the rightward (blue lines) in a period between – 5 and 30 ms. On the other hand, an ANOVA for the right eye’s velocity identified the reverse difference, i.e., larger variance in the rightward saccades than the leftward from 0 to 10 ms. Taken together, the abducting eye exhibited larger variance relative to the adducting eye. The latencies of the abducting-adducting effect are denoted by black square brackets. Also, in both eyes, the ANOVAs identified significantly larger variance in the Go/NoGo sessions (dashed lines) than the Go-Only sessions (solid lines) (left eye: between – 10 and 0 ms and between 10 and 30 ms; right eye: between 5 and 20 ms; green square brackets), and larger variance near the end of leftward saccades (red lines) relative to the rightward saccades (blue lines) (left eye: between 60 and 70 ms, right eye: between 50 and 65 ms). To see the effect of abducting-adducting and the cognitive load, see Supplementary Fig. 3A and B in Additional file 3.

	The onsets of significant differences are shown linked between the eyes in Fig. 5C. The abducting-adducting effect (black line) and the cognitive load effect (green line) were earlier in the left eye than the right eye, though the leftward-rightward effect was identified earlier in the right eye relative to the left eye.

	Results of statistical tests for the variance of position are shown in Supplementary Fig. 4 in Additional file 3. ANOVAs on positions found significantly larger variance for the abducting saccades than the adducting ones, in the Go/NoGo sessions than the Go-Only sessions, and on the 2^nd^ day than the 1^st^ day in both eyes. The leading eyes based on these effects were not consistent.

### Joint probability distribution

**Figure 6:**
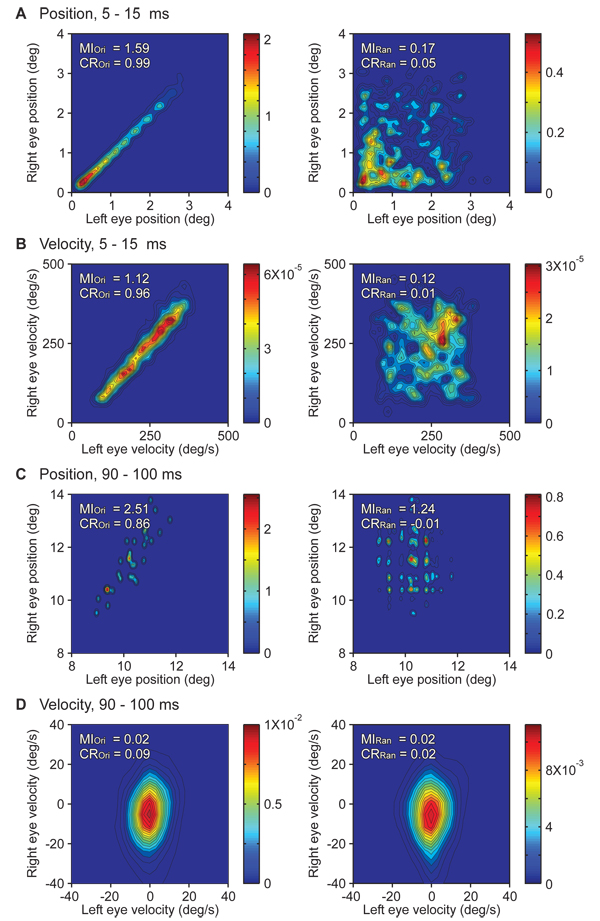
**Examples of joint probability density distributions, used for computation of mutual information (MI).** The distributions were estimated by applying a Gaussian kernel to: **A.** Eye position samples between 5 and 10 ms from saccade initiation; **B.** Eye velocity samples between 5 and 10 ms; **C.** Eye position samples between 90 and 100 ms; **D.** Eye velocity samples between 90 and 100 ms. The smoothing length of the kernel was optimized based on the samples by the likelihood cross validation method. The distributions are for a leftward saccade, Go/NoGo session on the 1^st^ day. Left panel: The samples were paired between the eyes in each trial with no delay. The MI of the original paired samples (MI_Ori_) and its correlation coefficient (CR_Ori_) are noted on the top left of each panel. Right panel: The pairs were randomized among trials within the time window of 11 ms. Because of the finite sample size (330 pairs). the MI of randomized samples (MI_Ran_) was often significantly larger than zero in spite of the randomization procedure. The MI_Ran_ was used to estimate the overestimation of relationship. The CR_Ori_ did not overestimate the relationship as shown by the correlation coefficient of the randomized samples (CR_Ran_). The probability densities are indicated by the colors, as specified in the color scales. The units of velocity are (deg/s)^-2^ and position deg^-2^.

	Representative joint probability distributions, used for MI analysis, are shown in Fig. 6 for the leftward saccades in the Go/NoGo sessions on the 1^st^ day. The eyes’ positions (Fig. 6A, left panel) and velocities (6B, left panel) were obtained between 5 and 15 ms. Here, we refer to the center of this time window as latency. The distributions indicate proportional relationships between the eyes’ movements, producing the high MI_Ori_ of 1.59 for velocity and 1.12 for position. In the right panels the relationships were removed by a randomization of the paired samples, which dramatically decreased the MI_Ran_. However, MI_Ran_ did not vanish completely, demonstrating the overestimation due to the limited sample size (330 pairs). In contrast, since CR analysis does not give an overestimation, the CR of randomised samples (CR_Ran_) became nearly zero.

	Figure 6C left panel shows a probability distribution of position in the period between 90 and 100 ms. The probabilities were concentrated on particular eye positions, which is a reflection of stable fixation during the analysis time window (11 ms) in each trial after the end of the saccade. The variance for the specific point of fixation of the eye positions is reflected in a trial variance when the eye is at the fixating position. The proportional relationship of the variance produced the high MI_Ori_ of 2.51. However, its high MI_Ran_ of 1.24 (Fig. 6C, right panel) indicates a large overestimation of the MI_Ori_ and the need for a correction method, i.e., a subtraction of MI_Ran_. While the eyes’ positions exhibited a strong proportional relationship, the probability distribution for velocity showed little relationship (Fig. 6D, left panel; MI_Ori_ = 0.02), and thus was not very different from the distribution of the randomized samples (Fig. 6D, right panel) as the probability densities for both had a normal distribution.

### Mutual information

**Figure 7:**
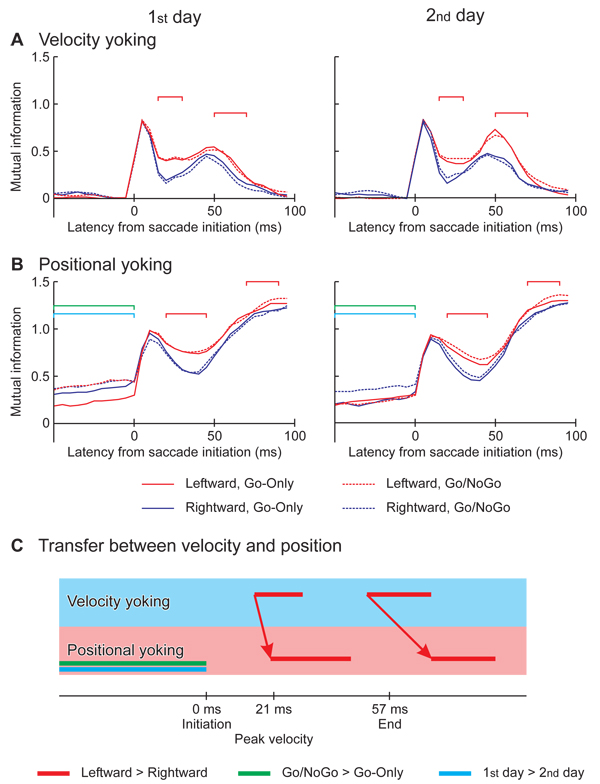
**Time courses of MI:A.** Between the left and right eyes’ velocities; **B.** Between the left and right eyes’ positions. The samples were paired between the eyes without delay relative to each other. The overestimation of MI was corrected by the difference between the MI_Ori_ and the mean MI obtained from 100 sample randomizations (): . MI_Cor_ is averaged among all subjects for the saccade toward the left (red lines) and right (blue lines) in the sessions of Go-Only (solid lines) and Go/NoGo (dashed lines), on the 1^st^ day (left panels) and 2^nd^ day (right panels). Horizontal axes are the latency from the saccade initiation. The latencies of significantly stronger relationship are denoted by red square brackets for the leftward saccade relative to the rightward, by green square brackets for the saccades in the Go/NoGo sessions than the Go-Only except for the leftward saccade on the 2^nd^ day, and by cyan square brackets for the saccades on the 1^st^ day than the 2^nd^ day except for the leftward saccade in the Go-Only session. The significant differences were revealed by ANOVA factoring the saccade direction, the session type, the experiment day and the subject (p < 0.05 for 10 ms or more). **C.** Time courses comparisons between the velocity MI_Cor_ and the positional MI_Cor_. The red arrows that link the red horizontal bars suggest causal relationship between the velocity MI_Cor_ and positional MI_Cor_ with respect to the significant difference between the leftward and rightward saccades. The green horizontal bar indicates the latency of significant cognitive load effect, and the cyan one indicates that of significant day effect, obtained only for the positional MI_Cor_.

The time courses of MI are shown for eye velocities in Fig. 7A and for positions in 7B. Averages among all subjects are shown for each experimental condition. MI was computed without the delay between the eyes. The velocity MI started to increase at 5 ms before the saccade initiation and exhibited its primary peak at 5 ms after the initiation, and then decreased. The velocity MI increased again at the latencies between 20 and 35 ms depending on the experimental conditions, and exhibited its secondary peak before the end of the saccade. On the other hand, the time courses for positional MI lagged behind the velocity MI. They increased at 0 ms and showed a peak at 10 ms. After a decrease, the positional MI started to increase again at the latencies between 35 and 50 ms depending on the conditions. These results suggested that positional yoking was a result of the velocity yoking.

An ANOVA using each time slice found that the velocity MI was significantly higher for the leftward saccades (Fig. 7A, red lines) than the rightward ones (blue lines) in periods between 15 and 30 ms and between 50 and 70 ms (red square brackets). The positional MI was also higher for the leftward saccades than the rightward ones in periods between 20 and 45 ms and between 70 and 90 ms (Fig. 7B, red lines). When Japanease read a text written in their traditional style, the eyes move downward and return in an upward direction, while the eyes of Caucasians move rightward and return with a quick saccade to the left. Thus, we added one factor of race (Japanese and Caucasian) and applied a five-way ANOVA (direction × session type × day × race × subject), but no significant main effect or interaction of race was obtained (p > 0.1). The positional MI before the saccade initiation was significantly higher in the Go/NoGo sessions (dashed lines) than the Go-Only sessions (solid lines) except for the leftward saccades on the 2^nd^ day, and on the 2^nd^ day (right panel) than the 1^st^ day (left panel) except for the leftward saccades in the Go-Only sessions.

	The onsets of significant effects for saccadic direction are linked between the velocity and positional MIs by red arrows in Fig. 7C. The direction effect on the velocity MI led with respect to that on the positional MI, which suggests that the velocity yoking was “causing” positional yoking.

	The CR analysis found the two peaks for velocity yoking after the initiation and before the end of saccades (Supplementary Fig. 5A in Additional file 3), as did the MI analysis. However, for position (Supplementary Fig. 5B in Additional file 3), the CR analysis showed only the peak yoking after saccade initiation and failed to detect the increase in yoking following the peak velocity. The CR analysis for position also failed to detect the cognitive load effect before saccade initiation (Supplementary Fig. 5B in Additional file 3). The failure of CR analysis to detect changes in yoking strength identified by the MI analysis is evidence for nonlinear contributions in positional yoking. The results therefore highlight an advantage for MI analysis compared to CR analysis in general and for studying yoking in particular.

## Discussion

### Source of statistical interocular relationship

The variance of each eye’s velocity was large during the saccades (Fig. 5A and 5B). In each experimental condition the variance peaked soon after saccade initiation. In some conditions a second peak in variance was identified just before saccade end. Similarly the MI (computed for zero interocular delay) was high during the saccade and peaked after the initiation and before the end (Fig. 7A). The two results show that velocity variances of the eyes were statistically linked to each other even when no interocular delay was allowed for. Thus the variances of the eyes could be partially explained by neural activity in control structures of the oculomotor pathway with bilateral rather than monocular influence. This confirmed a previous study in which the size of the correlation coefficient between the eyes was more than 0.75 for peak velocity, saccade duration and saccade curvature [4]. However, it can not be the sole explanation for velocity variance. In the current study, while the cognitive load increased the eyes’ velocity variance (Go/NoGo vs. Go-Only in Fig. 5A and 5B), it did not increase the velocity MI (Fig. 7A). This result suggests that the changes in cognitive load may alter neural processes in areas where oculomotor influence is either monocular, or at least weakly binocular. There could be several responsible structures in the neural control pathway. A previous study from our group using identical saccade task suggested that neural activity in the brainstem and the flocculus might be responsible. An increase in linkage between these areas just before and in the early part of the saccade was identified for the task with higher cognitive load in this previous MEG study [7]. 

The increase of velocity MI, after initiation and before the end of the saccade, led those of positional MI (Fig. 7A and 7B). Also the significant influence of saccade direction was identified for velocity MI before positional MI (Fig. 7C). These results suggested that positional yoking was caused by velocity yoking. This could be a consequence of the analysis method used since position was simply a temporal integration of velocity, the saccades being in the horizontal direction [6]. However CR analysis failed to associate the increase of velocity yoking before the saccadic end with that of positional yoking (Supplementary Fig. 5 in Additional file 3). These results therefore highlight the advantages of MI analysis over CR analysis.

	Before the initiation of saccades, the positional MI was significantly higher in the Go/NoGo sessions relative to the Go-Only sessions, and on the 1^st^ day compared to the 2^nd^ day (Fig. 7). Similarly, the positional fluctuations of the eyes were significantly large for the Go/NoGo sessions on the 1^st^ day (Supplementary Fig. 3C and D and Supplementary Fig. 4 in Additional file 3). Taken together, the larger variance of eye position could be a reflection of binocular neural control processes rather than at a monocular level, exerting an influence on the oculomotor system even before saccade initiation. The larger variance of position could possibly reflect a longer latency of saccade initiation for the Go/NoGo sessions on the 1^st^ day (Fig. 3A), rather than the cognitive load or a practice effect. Alternatively it may be a consequence of a more complex preparation sequence that reaches the ocular muscles and even prepares their state before the final command for saccade execution. Evidence for such a mechanism is presented in another paper from our group in this volume.

### Laterality

The effects of saccade direction and practice on the average velocity were identified in the left eye before those of the right eye (Fig. 4C). The large variance in velocity in the abducting saccades in Go/NoGo sessions was also found in the left eye prior to the right eye (Fig. 5C). Recalling the dominant eye was the right in all subjects, it is not likely that the left eye guided the right eye. A more plausible explanation is that the left eye movements were easily modulated by the different experimental conditions, and thus the right eye movements compensated so that the two eyes exactly foveated the same object.

Consistent laterality differences in timing across subjects have not been found for horizontal saccades [10,13]. In the current study, both eyes showed longer latencies to peak velocity for leftward than for rightward saccades (Fig. 3B). We also found a higher velocity for leftward saccades relative to rightward ones (Fig. 4). There were also differences in MI for the binocular control of leftward saccades, i.e., stronger leftward yoking than for rightward saccades (Fig. 7). In the previous MEG study [7], Fig. 3, also carried out with right handed subjects and the same tasks, coherency of neural activation across trials in the frontal eye field (FEF) of the right hemisphere clearly increased after the initiation of leftward saccades, whereas both sides of the FEF showed only a small increase for rightward saccades. Further, in another MEG study from this laboratory on saccades in both waking and sleeping, it was found that the interaction between cortical and sub-cortical areas were more complicated for leftward relative to rightward saccades in the waking condition [14], [Fig. 10]. These different neural processes for left and right saccades might be a reflection of the differences we found in this study from the behavioral data between left and right saccadic movements. The laterality of saccade direction was not significantly different for the reading groups, suggesting that a cultural bias would not account for the difference between saccades to the left and right, also many Japanese now read books written in Japanese with a standard western left right format. A recent study [15] has shown that the left and right parietal regions exert different control on saccade direction a further point for the asymmetry of saccade genesis.

### Outlook

Failures in basic mechanisms integrating information from the two eyes lead to pathological conditions, for example small lesions in the brainstem, cerebellum, cortex or in the thalamus can all lead to disorders of eye movements. More recently eye movement disorders have been described in schizophrenia, depression [16] and dementia [17]. It is not yet known if these movement changes can have predictions for treatment response. Our long term goal is to develop new clinical methods for the detection of early oculomotor deficits that could be widely available and cheap. This goal is pursued by two research strands. The first research effort continues the work of our earlier studies using detailed tomographic analysis of MEG data to describe activity in the oculomotor system under different conditions [7,14]. As part of the same work we also study in detail how key parts of oculomotor system are activated in specific tasks, e.g. the frontal eye field [18]. We are using the MEG to relate activity in specific brain areas in the cortex, cerebellum and brainstem, to specific eye-movement tasks. In the second research strand we are using the knowledge about spatiotemporal brain activity patterns to develop biomarkers for normal and pathological activity. These biomarkers could rely on the detection of spatiotemporal patterns with EEG or values of parameters extracted from eye tracking measurements. Successful implementation of our research goals could then lead to widely available and relatively cheap clinical tools that can be part of routine screening. The clinical utility for early diagnosis could include problems not only in the oculomotor areas, but also in areas in the frontal lobe, cerebellum and brainstem where any general disturbances will also affect key structures of the oculomotor system.

## Conclusions

We have shown that mutual information analysis is successful in determining the detailed relational structure of eye movements, and this analysis has given information about the saccade that was not apparent in the individual velocity and position measurements. This new method could have clinical utility for the detection of early ocular muscular imbalance in a number of pathological conditions where failures occur in basic mechanisms integrating information from the two eyes.

## Competing interests

The authors declare that they have no competing interests.

## Authors’ contributions

MM performed the experiment, developed the mutual information analysis with the contribution of AAI, and carried out all analyses. MM and AAI designed the experiment together guided by an earlier MEG design used in a separate MEG experiment. AAI conceived the usage of mutual information analysis to measure eyes’ yoking, and organized whole of the study. PBCF and AAI formulated the physiological and cognitive discussion of the results with the contribution of MM. The manuscript was written initially by MM and revised by PBCF and AAI. All authors read and approved the final manuscript.

## Supplementary Material

Additional file 1 Click here for file

Additional file 2 Click here for file

Additional file 3 Click here for file

## References

[B1] Jones RK Lee DN Why two eyes are better than one: the two views of binocular vision' J Exp Psychol Hum Percept Perform 1981 7 1 30 40 10.1037/0096-1523.7.1.30 6452501

[B2] Collins CC Carlson MR Scott AB Jampolsky A 'Extraocular muscle forces in normal human subjects', Invest Ophthalmol Vis 1981 20 5 652 664 7216678

[B3] Erkelens CJ Sloot OB 'Initial Directions and Landing Positions of Binocular Saccades' Vision Research 1995 35 23-24 3297 3303 10.1016/0042-6989(95)00077-R 8560800

[B4] Bains RA Crawford JD Cadera W Vilis T 'The Conjugacy of Human Saccadic Eye-Movements' Vision Research 1992 32 9 1677 1684 10.1016/0042-6989(92)90160-K 1455739

[B5] Shannon CE 'The mathematical theory of communication' The Bell System Technical Journal 1948 27 379 423

[B6] Tweed D Vilis T 'Implications of Rotational Kinematics for the Oculomotor System in 3 Dimensions' Journal of Neurophysiology 1987 58 4 832 849 3681398 10.1152/jn.1987.58.4.832

[B7] Ioannides AA Fenwick PB Liu L 'Widely distributed magnetoencephalography spikes related to the planning and execution of human saccades' J Neurosci 2005 25 35 7950 7967 10.1523/JNEUROSCI.1091-05.2005 16135752 PMC6725466

[B8] Oldfield RC 'Assessment and Analysis of Handedness - Edinburgh Inventory' Neuropsychologia 1971 9 1 97 113 10.1016/0028-3932(71)90067-4 5146491

[B9] Silverman BW 'Density Estimation for Statistics and Data Analysis' 1986 Chapman & Hall

[B10] Collewijn H Erkelens CJ Steinman RM 'Binocular co-ordination of human horizontal saccadic eye movements' J Physiol 1988 404 157 182 3253429 10.1113/jphysiol.1988.sp017284 PMC1190820

[B11] Collewijn H 'Interocular timing differences in the horizontal components of human saccades' Vision Res 2001 41 2526 3413 3423 10.1016/S0042-6989(01)00047-5 11718783

[B12] Smith KU Schmidt J Putz V 'Binocular coordination: feedback of synchronization of eye movements for space perception' Am. J Optom. Arch. Am. Acad. Optom 1970 47 9 679 689 5273340 10.1097/00006324-197009000-00003

[B13] Honda H 'Idiosyncratic left-right asymmetries of saccadic latencies: examination in a gap paradigm' Vision Res 2002 42 11 1437 1445 10.1016/S0042-6989(02)00050-0 12044750

[B14] Ioannides AA Corsi-Cabrera M Fenwick PB del Rio PY Laskaris NA Khurshudyan A Theofilou D Shibata T Uchida S Nakabayashi T Kostopoulos GK 'MEG tomography of human cortex and brainstem activity in waking and REM sleep saccades' Cereb. Cortex 2004 14 1 56 72 10.1093/cercor/bhg091 14654457

[B15] Kapoula Z Yang Q Coubard O Daunys G Orssaud C 'Role of the posterior parietal cortex in the initiation of saccades and vergence: right/left functional asymmetry' Ann 2005 1039 184 197 10.1196/annals.1325.018 15826973

[B16] Harris MS Reilly JL Thase ME Keshavan MS Sweeney JA 'Response suppression deficits in treatment-naïve first-episode patients with schizophrenia, psychotic bipolar disorder and psychotic major depression' Psychiatry Res 2009 170 23 150 6 10.1016/j.psychres.2008.10.031 19906441 PMC2792232

[B17] Crutcher MD Calhoun-Haney R Manzanares CM Lah JJ Levey AI Zola SM 'Eye tracking during a visual paired comparison task as a predictor of early dementia' Am J Alzheimers Dis Other Demen 2009 24 3 258 66 10.1177/1533317509332093 19246573 PMC2701976

[B18] Ioannides AA Fenwick PBC Pitri E Liu L 'A step towards non-invasive characterization of the human frontal eye fields of individual subjects' Nonlinear Biomedical Physics 2010 4 Suppl1 10.1186/1753-4631-4-S1-S11 PMC2880797 20522261

