# Supplementary material for: Interocular yoking in human saccades examined by mutual information analysis
Source: Nonlinear Biomed Phys. 2010 Jun 3;4(Suppl 1):S10. doi: 10.1186/1753-4631-4-S1-S10 (PMC2880796; doi:10.1186/1753-4631-4-S1-S10)
Supplement: Additional file 1 [file 1753-4631-4-S1-S10-S1.pdf]

# Supplementary materials

## Detection of saccade latencies

First, the initial consecutive time slices with eye speeds exceeding 10 deg/s for at least 40 ms were noted. The period of such consecutive time slice was defined as a saccade candidate duration. Next, the saccade candidate duration for the first time slice that was followed by a continuous increment of eye speed for at least 6 ms was found. Saccade initiation time was defined as this initial time slice. Saccade end time was defined as the first time slice after the end of the saccade candidate duration when the eye speed dropped below 10 deg/s. The time of peak-velocity was automatically detected within the range between the saccade initiation and the end latency. The latencies of these key events were determined for each trial by using averaged velocity between the eyes. After automatic detection, we visually validated the detected parameters.

## Optimized Gaussian kernel

For the mutual information (MI) analysis, a joint probability distribution of the left eye's velocity $x$ and the right eye's velocity $y$ was estimated using a Gaussian kernel, defined as

$$\hat{P}(x,y) = \frac{1}{N \cdot h_x \cdot h_y} \sum_{k=1}^{N} \frac{1}{2\pi} \exp\left[ -\frac{(x - X_k)^2}{2 \cdot h_x^2} - \frac{(y - Y_k)^2}{2 \cdot h_y^2} \right], \tag{S1}$$

where $X_k$ and $Y_k$ denote the velocity samples from the left and right eyes and $N$ is the number of samples. $h_x$ and $h_y$ represent the smoothing lengths of the Gaussian kernel. Optimal smoothing lengths depend on sample size and distribution. The dispersion of $X_k$

can be different from that of $Y_k$; thus, the optimal smoothing length should also be different between the eyes. However, we wanted to reduce the number of free parameters to be optimized in order to reduce the computational load. To do this, we first normalized the samples in relation to their standard deviation as follows:

$$Xn_k = \frac{X_k - \overline{X}}{SD_X}, \quad Yn_k = \frac{Y_k - \overline{Y}}{SD_Y}, \tag{S2}$$

where $\overline{X}$ and $SD_X$ are the mean and standard deviation of samples $X_k$, and similarly for $Y_k$. After this normalization, the Gaussian kernel estimator of probability density is given as follows:

$$\hat{P}(x,y) = \frac{1}{Nh^2}\sum_{k=1}^{N}\frac{1}{2\pi}\exp\left[-\frac{(x - Xn_k)^2 + (y - Yn_k)^2}{2\cdot h^2}\right], \tag{S3}$$

where $h$ represents the common smoothing length between the eyes. The Gaussian kernel for the eyes positions was also defined similarly.

The smoothing length was optimized by a likelihood cross validation method (Silverman, 1986). The difference between the estimated probability density $\hat{P}(x,y)$ and the true one $P(x,y)$ is quantified as the information distance, defined as

$$ID(h) = \iint P(x,y)\ln\left\{P(x,y)/\hat{P}(x,y)\right\}dxdy\,. \tag{S4}$$

*ID(h)* contains the function $P(x,y)$ that we are seeking. However, we can minimize *ID(h)* without knowing $P(x,y)$ because the optimal smoothing length maximizes the score function

$$CV(h) = \frac{1}{N}\sum_{k=1}^{N}\ln\hat{P}_{-k}(Xn_k, Yn_k)\,, \tag{S5}$$

where $\hat{P}_{-k}(Xn_k, Yn_k)$ is the probability density at $Xn_k$ and $Yn_k$, estimated by using all samples except the $k$th sample. We sought the value $h$ that maximized the score function *CV(h)*. See Supplementary Fig. 1D for the dependence of optimal smoothing length on the sample size and on the probability distribution.

The joint probability distribution estimated by the optimized kernel was used for the MI analysis.

## Simulation

To show quantitatively how the optimized kernel works for the MI estimation, we simulated our method using computer-generated samples. In our simulation, sets of paired samples were generated according to a given probability distribution given by:

$$P(x, y) = \frac{1}{2\pi\sqrt{1 - C^2}} \exp\left(-\frac{x^2 + y^2 - 2\,C \cdot x \cdot y}{2\,(1 - C^2)}\right), \qquad \text{(S6)}$$

where $C$ is the product-moment correlation coefficient between $x$ and $y$. The MI value of the distribution was varied between 0 and 1 in steps of 0.1 by using different $C$ values. When the MI value was zero, obtained by setting $C$ at zero, the distribution became the well-known bivariate normal distribution. The MI value estimated from the samples was compared with the true MI value, which was computed directly from the given probability distribution. The sample size of each MI calculation was 50, 100, 200, or 400. The computer generation of samples was repeated 100 times; thus, we obtained 100 MI values for each given distribution and sample size.
