# Supplementary material for: Interocular yoking in human saccades examined by mutual information analysis
Source: Nonlinear Biomed Phys. 2010 Jun 3;4(Suppl 1):S10. doi: 10.1186/1753-4631-4-S1-S10 (PMC2880796; doi:10.1186/1753-4631-4-S1-S10)
Supplement: Additional file 2 [file 1753-4631-4-S1-S10-S2.pdf]

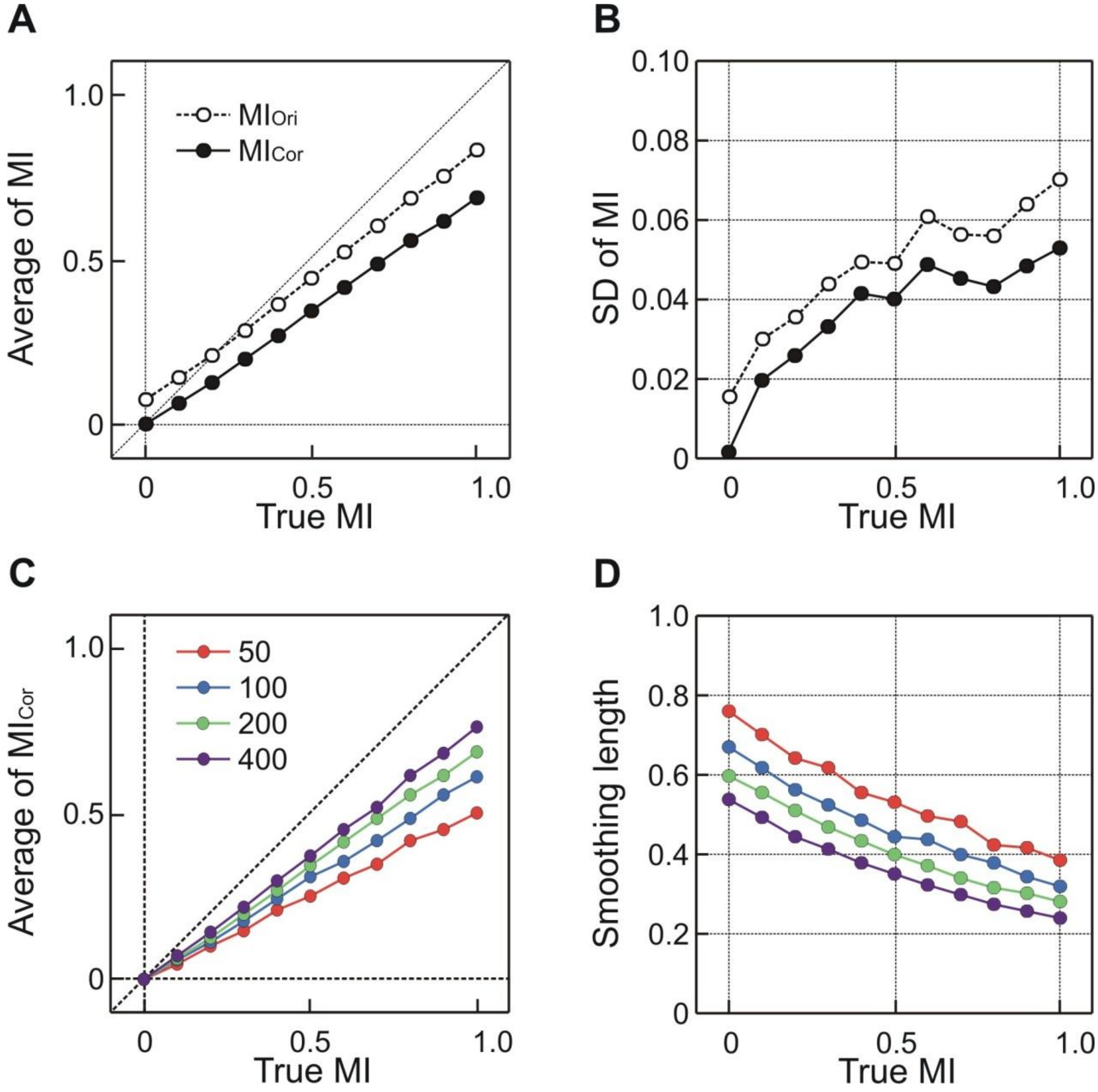

Supplementary Figure 1. Simulation of MI analysis using computer-generated samples. The true MI values, which were obtained directly from the given probability distributions, are shown on the horizontal axes. **A.** Averages of MI from original samples (MI_{Ori}, open symbol on dashed line) and corrected MI (MI_{Cor}, filled symbol on solid line), defined as the difference between the MI_{Ori} and the mean MI obtained from 100 sample randomizations ($\overline{MI}_{Ran}$): $MI_{Cor} = MI_{Ori} - \overline{MI}_{Ran}$. The samples size in each MI computation was 200. This sample set was repeatedly generated 100 times, producing 100 MI values. The average among the 100 MI values is shown. **B.** SD of the 100 MI

values. **C.** Averages of MI_{Cor} for difference sample size (50, 100, 200 and 400), as noted by different colors. The result demonstrated the influence of sample size on the MI estimation. **D.** Averages of optimized smoothing length by the likelihood cross validation method. The result demonstrated that the optimal smoothing length was different for the different sample size. Also the optimal length was different among the different true MI values, as expected from the different probability distribution for the different true MI values.
