# Supplementary material for: Interocular yoking in human saccades examined by mutual information analysis
Source: Nonlinear Biomed Phys. 2010 Jun 3;4(Suppl 1):S10. doi: 10.1186/1753-4631-4-S1-S10 (PMC2880796; doi:10.1186/1753-4631-4-S1-S10)
Supplement: Additional file 3 [file 1753-4631-4-S1-S10-S3.pdf]

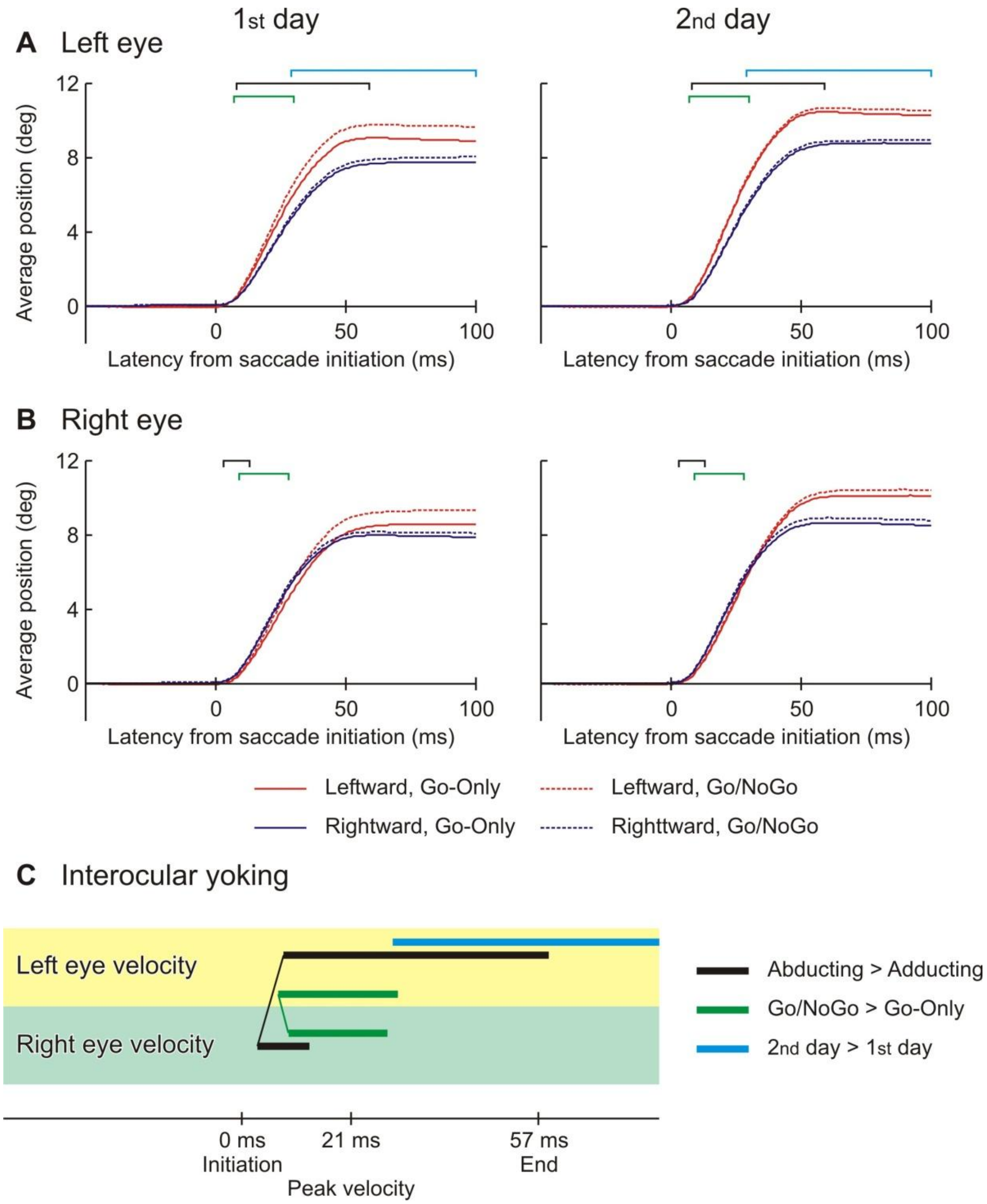

Supplementary Figure 2. Dependency of averaged eye positions on experimental conditions: **A.** Left eye; **B.** Right eye. Averages for all subjects are shown for saccade toward the left (red lines) and right (blue lines) for the sessions of Go-Only (solid lines) and Go/NoGo (dashed lines), on the 1st day (left panels) and 2nd day (right panels). Positive deflections (vertical axis) denote eye movements towards saccadic

target. Horizontal axes are the latency from saccade initiation. The significant difference between abducting and adducting saccade latencies of eye position shift from the fixation point to the target are denoted by black square brackets, by cyan square brackets for the significant difference between the 1st and 2nd days, and by green square brackets for the significant difference between the Go-Only and Go/NoGo sessions. The significant differences were revealed by ANOVA factoring the saccade direction, the session type, the experiment day and the subject ($p < 0.05$ for 10 ms or more). Note the differences between the saccade directions after the saccade end were not significant because of large individual variances. **C.** Time courses comparisons between the eyes based on the significant differences. The latencies of significant dependency on saccade direction are indicated by the black horizontal bars. The onsets of the significant dependency are linked between the eyes by a thin line, showing eye yoking. The green horizontal bars linked by a thin line indicate the yoking found based on the significant effect of cognitive load. The cyan horizontal bar shows the latency of the significant day effect.

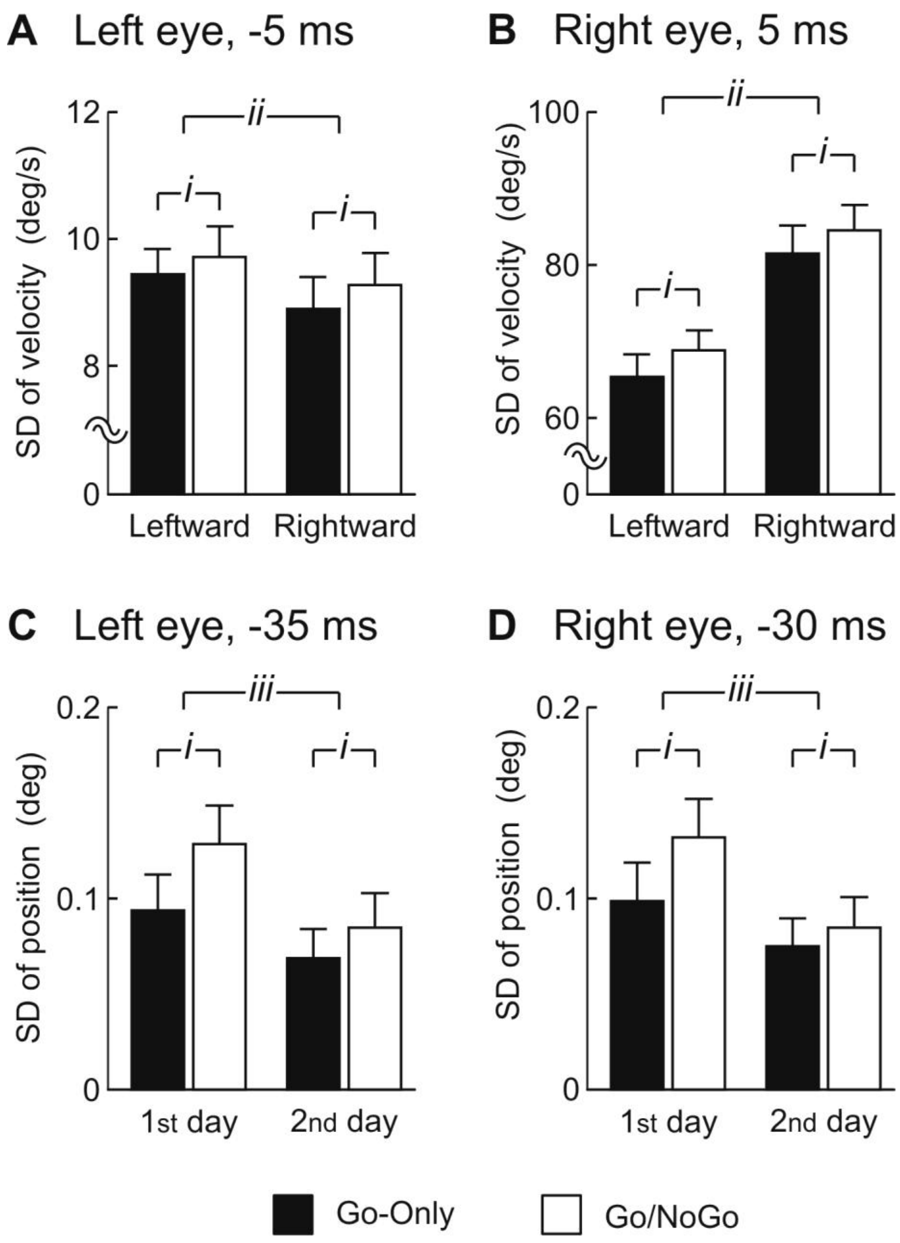

Supplementary Figure 3. Trial- by-trial variance of: **A.** Left eye velocity at –5 ms from saccade initiation; **B.** Right eye velocity at 5 ms. Standard deviations (SD) within each subject and experimental condition are averaged among all subjects, and also between the 1st and 2nd days because of no significant difference between the days. Error bars represent ± 1 SE. Significant differences were: *i*. Between Go-Only and Go/NoGo sessions; *ii*. Between leftward and rightward saccades, revealed by ANOVA factoring the

saccade direction, the session type, the experiment day and the subject ($p < 0.05$). **C.** SD of left eye position at –35 ms; **D.** SD of right eye position at –30 ms. SDs are averaged among all subjects, and also between the leftward and rightward saccades because of no significant difference between the directions. The ANOVA revealed significant differences: *i.* Between the Go-Only and Go/NoGo sessions; *iii.* Between the 1st and 2nd days.

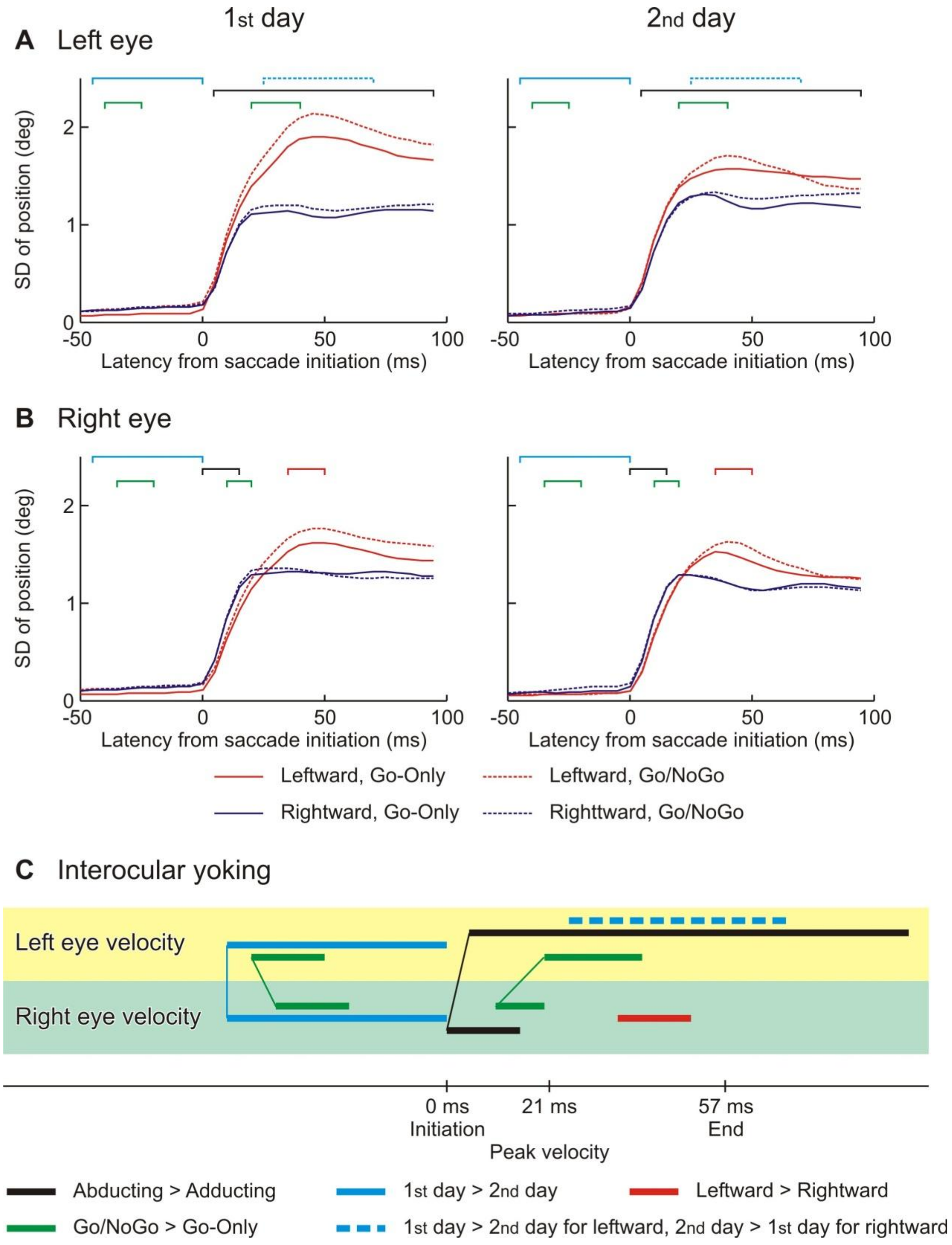

Supplementary Figure 4. Dependency of trial-by-trial variances of eye position on experimental conditions: **A.** Left eye; **B.** Right eye. Standard deviation within each subject is averaged among all subjects for the saccade toward the left (red lines) and right

(blue lines) in the sessions of Go-Only (solid lines) and Go/NoGo (dashed lines), on the 1st day (left panels) and 2nd day (right panels). Horizontal axes are the latency from saccade initiation. The latencies of significantly larger variances are denoted by black square brackets for the abducting saccade relative to the adducting, by red square brackets for the leftward saccade relative to the rightward, by green square brackets for the saccades in the Go/NoGo sessions than the Go-Only, and by cyan square brackets for the saccades on the 2nd day than the 1st day. The dashed square bracket in cyan indicates a significantly larger variance of the leftward saccade on the 1st day than 2nd day and with that of the rightward saccade on the 2nd day than the 1st day. The significant differences were revealed by ANOVA factoring the saccade direction, the session type, the experiment day and the subject ($p < 0.05$ for 10 ms or more). For a viewable difference between the Go/NoGo and Go-Only sessions, see Supplementary figure 3C. **C.** Time courses comparisons between the eyes based on significant differences. The latencies of significantly larger variance of the abducting saccade than the adducting are indicated by black horizontal bars. The onsets of the significant difference are linked between the eyes by a thin line, showing eye yoking. Linked horizontal bars in green indicate the latencies of yoking found based on the significantly larger variance in the Go/NoGo sessions than the Go-Only, and that in cyan based on the significantly larger variance on the 1st day than the 2nd day. Other bars are as noted in the figure text.

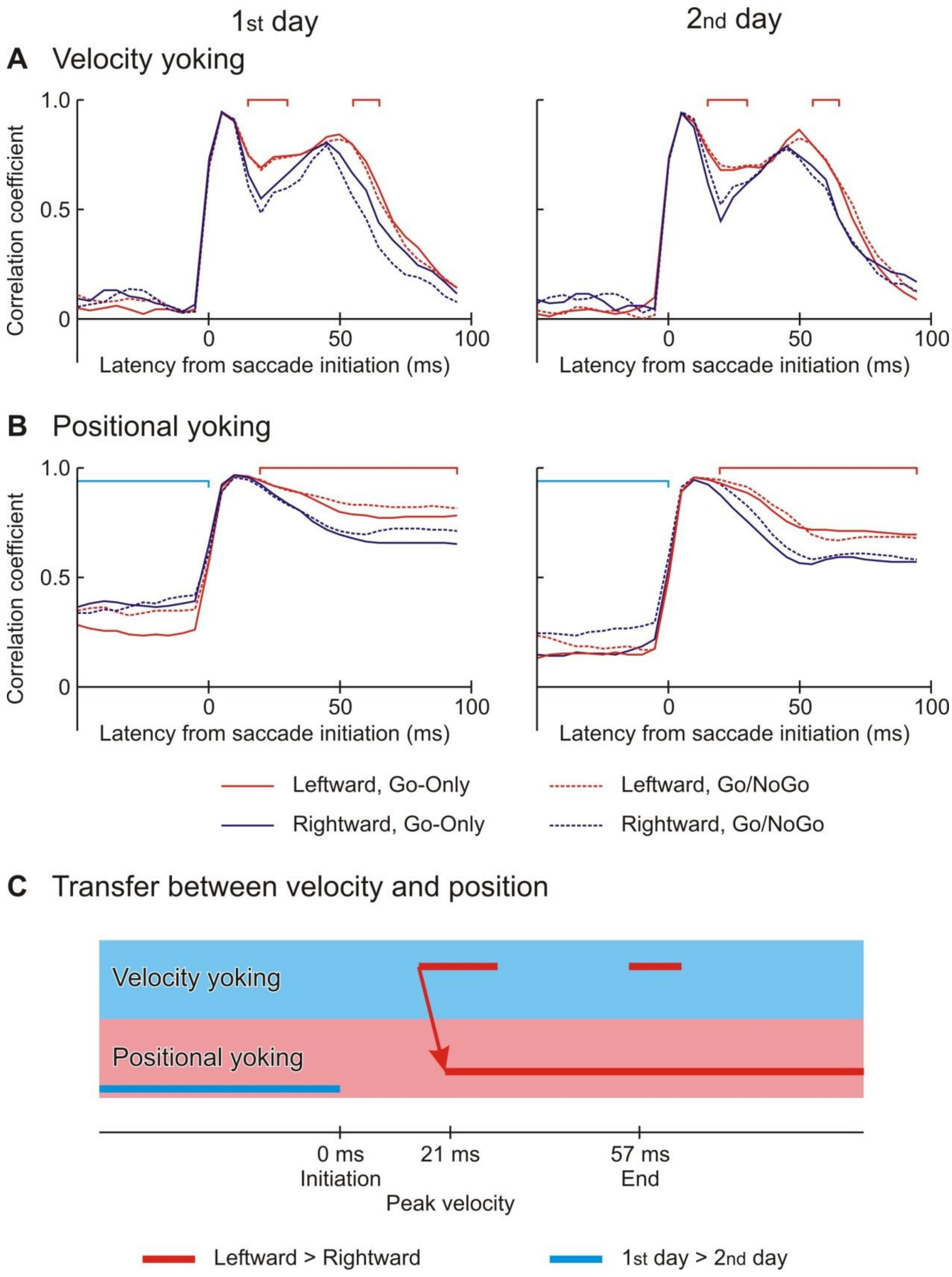

Supplementary Figure 5. Time courses of correlation coefficient (CR): **A.** Between the left and right eye velocities; **B.** Between the left and right eye positions. The samples were paired between the eyes without delay relative to each other. CR is

averaged among all subjects for the saccade toward the left (red lines) and right (blue lines) in the sessions of Go-Only (solid lines) and Go/NoGo (dashed lines), on the 1st day (left panels) and 2nd day (right panels). Horizontal axes are the latency from the saccade initiation. The latencies of significantly stronger relationship are denoted by red square brackets for the leftward saccade relative to the rightward, and by cyan square brackets for the saccades on the 1st day than the 2nd day. The significant differences were revealed by ANOVA factoring the saccade direction, the session type, the day and the subject ($p < 0.05$ for 10 ms or more). **C.** Time courses comparisons between the velocity CR and the positional CR. Linked Red horizontal bars linked by arrow indicate the causal relationship between the velocity CR and the positional CR, found based on the significant difference between the leftward and rightward saccades. The cyan horizontal bar indicates the latency of significant day effect. Note that, unlike the MI analysis, the significant effect of cognitive load and the increase of yoking after the saccade end were not found by the CR analysis.
